# Distress Screening in Chronic Disease: Essential for Cancer Survivors

**Published:** 2014-03-01

**Authors:** Lorie Petty, Joanne Lester

**Affiliations:** From Division of Nursing, James Cancer Hospital & Solove Research Institute, The Ohio State University, Columbus, Ohio; Department of Psychology and Comprehensive Cancer Center, The Ohio State University, Columbus, Ohio

## Abstract

Distress is a psychological state that is often observed in patients with chronic disease. Many cancers are considered chronic in nature, with patients experiencing long, disease-free states and intervals of metastatic disease. Distress can negatively affect the biopsychosocial balance in cancer survivors and impede their progress along the cancer trajectory. Distress can also affect medical and psychological outcomes and hinder advancement into long-term survivorship. Distress may contribute to disease progression, although despite research findings, health-care providers seldom screen for indications of persistent or unresolved distress. This article discusses research findings related to the prevalence of distress in multiple chronic diseases. Validated instruments used to screen for distress in cancer survivors, such as the Distress Thermometer and symptom checklist from the National Comprehensive Cancer Network, are reviewed. With the availability of brief and concise instruments to screen for distress, providers have the ability to provide holistic and comprehensive care for cancer survivors. The overall financial impact of cancer-related distress is understudied, although similar psychological studies indicate that prevention or elimination of distress is beneficial. Cancer is a lifelong, chronic disease; patients have ongoing needs and varied sources of distress. As the number of cancer survivors exponentially increases, their psychosocial needs will likewise expand.

Distress can be defined as a psychological state exacerbated by physical symptoms, interpersonal challenges, psychological symptoms, social issues, and/or existential concerns. It is a common problem in patients with cancer and other chronic diseases, although screening, assessment, and interventions are not routinely performed (Bultz & Carlson, 2006). Prolonged exposure to stress can have a negative effect on quality of life and manifest a host of physical symptoms. Studies have demonstrated that stress may also contribute to disease progression (Lillberg et al., 2003). This article highlights the prevalence of distress in chronic disease, screening methods and frequency, and applications of the transdisciplinary team in the continuing care of cancer survivors.

## Stress in Chronic Disease

Stress is a common symptom in chronic disease states, as illustrated below in a discussion of several common comorbidities. While stress has been studied in patients with cancer, it is uncommon to observe practice patterns that account for, intervene in, or follow up on the distress experienced by the cancer patient and his or her family members. In addition, patients with cancer often suffer stress related to comorbidities; several of the most common are discussed below. Therefore, it is important to understand the stress/distress within these entities and the possible overlap or exacerbation that can occur with the addition of a cancer diagnosis or a rediagnosis with metastatic disease.

## Rheumatoid Arthritis

Rheumatoid arthritis (RA) is a chronic and painful illness that can cause patients to lose daily functional abilities. Patients experience disruptions in multiple facets of their lives, including work, school, social, and family contexts. As a result of these alterations, patients can experience a high rate of psychological distress. A Turkish study examined 117 patients with RA using the Hospital Anxiety and Depression Scale (HADS). Results indicated that 58.1% of patients scored above the cutoff point for anxiety, while 55.6% scored above the cutoff point for depression (Dirik & Karanci, 2010). The authors noted that these levels were higher than those previously found in RA studies and attributed these findings to the lower socioeconomic status of the study sample (Dirik & Karanci, 2010).

Another study that utilized the HADS noted that psychological distress in RA patients significantly correlated with levels of pain (*p* < .05) and functioning (*p* < .05; Norton, Sacker, Young, & Done, 2011). The authors emphasized that clinicians should address psychological distress in RA patients, especially when somatic symptoms increase (Norton et al., 2011).

A third study of RA patients (N = 230) demonstrated that psychological distress was significantly correlated with both functional disability and perceptions of greater symptomatology (van Os, Norton, Hughes, & Chilcot, 2012). In another study of 71 patients with RA, the effects of a mindfulness-based program were tested. It was noted that 32.4% of the participants (N = 71) had psychological distress at baseline (Zangi et al., 2012).

## Diabetes

Diabetes is another chronic disease that is commonly associated with increased psychological distress. Diabetic patients are likely to have depression as well as decreased adherence to either insulin or oral agent management. In addition, these patients often have poor metabolic control, increased complication rates, alterations in quality of life, increased disability, lower productivity, higher use of health care and resultant costs, and ultimately, increased risk of death (Egede & Dismuke, 2011). Participants with types 1 and 2 diabetes who demonstrated increased levels of psychological distress were associated with poor glycemic control and suboptimal medication adherence (Egede & Dismuke, 2011). The presence of serious psychological distress (SPD), a 12-month Diagnostic and Statistical Manual of Mental Disorders (5th ed.) disorder, and a Global Assessment of Functioning score of less than 60 (scale of 0 to 100) provided a profile that was twice as prevalent in individuals with diabetes as in those without diabetes (Egede, & Dismuke, 2011). Therefore, it was determined that SPD has a marked negative impact on diabetic care and outcomes (Egede & Dismuke, 2011).

In a survey conducted in California, patients with diabetes and SPD were more likely to report insufficient levels of physical activity and greater levels of smoking than diabetic patients without SPD (Shin, Chiu, Choi, Cho, & Bang, 2011). Results of this study prompted researchers to consider a study with interventions to treat SPD and an examination of the effect on wellness outcomes in persons with diabetes.

## Other Inflammatory Conditions

The markers of other chronic inflammatory conditions can be correlated with similar inflammatory markers in cancer. These diseases are indicative of processes that predicate one to develop a host of various disorders secondary to intrinsic inflammation. Patients with psoriasis and atopic eczema experienced elevated levels of distress and scored significantly higher on the HADS than the control group (Mizara, Papadopoulos, & McBride, 2011). The mean scores for anxiety and depression within each disorder suggested probable mood disorder. No significant differences in HADS scores (*p* < .05) were found between patients with chronic skin disease and diabetic patients, which suggested that the psychological distress stems at least in part from the chronic nature of the disease (Mizara et al., 2011).

A study of 564 patients with Rome-positive irritable bowel syndrome (IBS) and 126 patients with inflammatory bowel disease (IBD) measured psychological distress with the Symptom Checklist 90-R (SCL-90-R). Psychological distress was found to have a stronger effect on health-related quality of life (-0.51 and -0.48 for IBS and IBD, respectively) than gastrointestinal (GI) symptoms (-0.25 and -0.28; Naliboff et al., 2012). In order to improve and maintain health-related quality of life in GI patients, psychological distress must be addressed (Naliboff et al., 2012).

Another study that used the SCL-90-R noted that patients with restless leg syndrome had evidence of elevated levels of psychological distress, particularly those with more severe symptoms (*p* = .013; Scholz et al., 2011). Similar to aforementioned studies, the authors highlighted the importance of distress assessment in order to improve outcomes (Scholz et al., 2011).

## Cardiopulmonary Disease

Psychological distress was found to have a negative effect on patients (N = 538) with coronary heart disease (CHD) and was a predictor of mortality in stable CHD patients following cardiac rehab (*p* < .0001). Depression appeared to have the largest impact on mortality outcomes, which highlighted the need to address depressive symptoms after completion of cardiac rehabilitation to improve overall survival (deSchutter, Lavie, & Milani, 2011).

In a study of 48 patients with chronic obstructive pulmonary disease (COPD) and their partners, high levels of psychological distress were observed (Meier, Bodenmann, Morgeli, & Jenewein, 2011). The partners’ own (negative) coping skills correlated negatively with their own physical (r = -0.34, *p* < .05) and psychological (r = -0.40, *p* < .05) quality of life, although a positive correlation was observed between the partners’ assessment of delegated dyadic coping (coping with stress within a couple) and environment-related quality of life (r = 0.51, *p* < .01). Overall, distress was positively correlated with dyadic coping. The authors emphasized that early detection of distress in both the patient and the partner could help improve dyadic coping (Meier et al., 2011) as well as physical and psychological outcomes related to COPD.

## Chronic Spine Disorders

Adult ambulatory patients (N = 149) with spine disorders categorized as degenerative, deformity, trauma, or tumor/infection were seen in clinic at the Department of Veterans Affairs and assessed for distress with the Distress and Risk Assessment Method (DRAM). Common to the sample were comorbid conditions and poorer health status. Psychological distress was observed in 80% of participants; of those, 43% were found to have severe psychological distress (Patton et al., 2012). Patients with severe distress reported higher levels of back pain (*p* = .036) as well as higher narcotic (*p* = .043) and antidepressant (*p* = .001) use as compared to the control group (Patton et al., 2011). Based on their study results, the authors recommended incorporation of routine assessment of psychological distress in patients with chronic back pain (Patton et al., 2012). Chronic back pain is a common symptom in cancer survivors and should be addressed in combination with other symptoms.

## Distress Screening in Cancer

The rate of distress screening in cancer patients remains low despite the option to utilize numerous reliable and valid, brief instruments. According to the Institute of Medicine, only 14% of 1,000 randomly selected members of the American Society of Clinical Oncology (ASCO) screened patients for distress (Jacobsen & Ransom, 2007). Additionally, only 8 of 15 National Comprehensive Cancer Network (NCCN)-designated centers screened for distress (Jacobsen & Ransom, 2007). Despite recommendations to routinely screen cancer survivors for distress as evidenced in the 2008 report from the Institute of Medicine (IOM), few providers and institutions followed the guidelines.

## NCCN’s Distress Thermometer

The NCCN’s Distress Thermometer (DT) is a brief self-report instrument that provides patient-reported screening data about distress that surrounds their cancer diagnosis and its impact on their psychological status (see Table below). With the familiar image of a thermometer (0 to 10 analog scale, with 0 = no stress and 10 = extreme stress; a score of 4 or more may be indicative of a moderate to high level of distress, and further assessment of the patient is indicated), patients can report their level of distress and its source with a list of common treatment-related difficulties (NCCN, 2012).

**Table 1:**
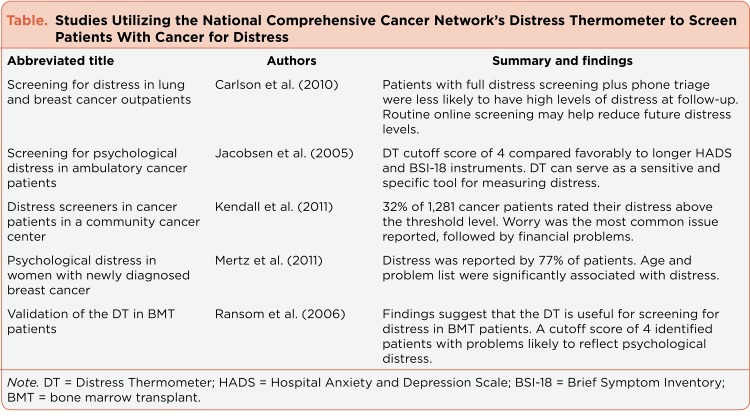
Table. Studies Utilizing the National Comprehensive Cancer Network’s Distress Thermometer to Screen Patients With Cancer for Distress

The DT has psychometric properties as demonstrated in multiple studies with the HADS and the Brief Symptom Inventory-18 (BSI-18). Accuracy was determined through comparison of the DT, the HADS, and the BSI-18; the receiver-operated characteristic curve analyses of the DT yielded under-the-curve estimates similar to the cutoff scores of the HADS (0.80) and the BSI-18 (0.78). Receiver-operated characteristic curve analyses also demonstrated optimal sensitivity and specificity with a DT cutoff score of 4 (Jacobsen et al., 2005).

In a study of 491 patients who prepared for bone marrow transplantation, the DT demonstrated significant correlations with the Center for Epidemiologic Studies–Depression Scale (CES-D) and the State-Trait Anxiety Inventory–State Version (STAIS-S). In this study, the DT exhibited a sensitivity ratio of 0.80 and a specificity ratio of 0.70 (Ransom, Jacobsen, & Booth-Jones, 2006). Overall, the single-item DT (i.e., analog distress thermometer only) compared well with other longer measures to assess psychological distress (Ransom et al., 2006). This study addressed the need for a brief, easily understood measure for distress.

Patients with newly diagnosed lung (n = 549) and breast (n = 585) cancer participated in a study that screened for emotional distress (Carlson, Groff, Maciejewski, & Builtz, 2010). These patients represented 89% of all new patients in the oncology practice under study (Carlson et al., 2010). Patients were randomized to one of three groups that incorporated use of the NCCN’s DT: (1) DT analog scale plus usual care; (2) DT analog scale, problem checklist, and usual care; or (3) DT analog scale, problem checklist, and follow-up telephone triage with referral to resources.

A high prevalence of distress was noted in 2/3 of the sample as indicated by a score of 4 or more on the DT analog scale. Seventy-five percent of the sample was retained for follow-up and reassessed 3 months later. Within the lung cohort that received full screening and triage (group 3), a 20% decrease in high levels of distress was noted at the 3-month follow-up visit. Within the breast cohorts, lower levels of distress were noted in groups 2 and 3. Referral to psychological services was the best predictor of decreased anxiety and depression in groups 2 and 3. This finding indicated that relaying improved assessment information to the practitioner resulted in more consults and comprehensive care, especially in the breast group (Carlson et al., 2010).

A study that utilized the DT instrument to assess distress in medical and radiation oncology cancer patients (N = 1,291) observed that 32% of patients reported distress above the threshold level (Kendall, Glaze, Oakland, Hansen, & Parry, 2011). In addition, 59% of participants indicated emotional concerns in the emotional subscale of the DT (Kendall et al., 2011). Patients were also provided a list of psychosocial providers; they could request for a referral to see one or more of the listed professionals. The cancer dietitian was the most frequently requested psychosocial referral. These findings indicate that there are unmet needs for psychosocial distress in cancer patients (Kendall et al., 2011).

A Danish study incorporated a DT cutoff score of 3 to indicate high levels of distress. The researchers observed that 77% of women with newly diagnosed breast cancer reported high levels of distress (Mertz et al., 2011). The most frequently reported sources of distress for these patients were worry and nervousness (Mertz et al., 2011). Women less than 50 years old reported higher levels of distress than women over 50 (Mertz et al., 2011).

## Economic Considerations

Little research providing financial evidence of the effect of psychosocial interventions on health-care utilization is available, specifically in cancer-related distress. As reviewed in multiple studies discussed previously, psychosocial distress is a significant problem that may occur in over one-half of cancer survivors (Carlson et al., 2010; Kendall et al., 2011; Mertz et al., 2011). Overall, it has been observed that timely treatment of emotional issues results in fewer outpatient visits to primary care providers and specialists (Carlson & Bultz, 2004).

A study that examined the impact of mental health symptoms on health-care expenditures over time recognized the potential effect of distress on patients with cancer (Pirraglia, Hampton, Rosen, & Witt, 2011). As psychological distress increased in patients, so did health-care expenditures (*p* < .001) and outpatient expenditures (*p* < .001). Covariates associated with higher levels of distress included older age, female gender, single marital status, racial minority, poverty, lack of insurance, lower education, rural status, and comorbid medical and psychological conditions (Pirraglia et al., 2011). These findings suggest that distress screening may improve financial concerns in health care and alleviate negative effects of distress (Pirraglia et al., 2011).

## Barriers to Distress Screening

Despite the evidence and recommendations for ongoing distress screening in cancer patients, improvements in transdisciplinary health care still need to occur. A study that screened for distress observed that 36.3% of 2,297 cancer patients had scores indicative of "psychiatric morbidity." Physicians misclassified these patients 34.7% of the time. This suggests that psychological and emotional illnesses in cancer patients are underrecognized (Fallowfield, Ratcliffe, Jenkins, & Saul, 2001).

Interviews with members of the transdisciplinary oncology health-care team noted that cancer care professionals understand the importance of detection of emotional distress in patients but are not as certain of individual roles and responsibilities. The physician or nurse seldom assumed responsibility to screen or treat distress, but instead expected the clinical nurse specialists to handle issues (Absolom et al., 2011). Oncologists and surgeons did not regard the responsibility of screening for and treating emotional distress to be an important aspect of their jobs. Another major barrier to the management of distress was the lack of a transdisciplinary referral pathway (Absolom et al., 2011), although the NCCN guidelines (2013) outline various pathways for the clinician and consultation of transdisciplinary practices.

In another survey study of oncology practices, 448 of 965 of invited oncologists responded. These oncologists estimated that over one-third of their patients experienced psychosocial distress, although only 50% of oncologists indicated that they had available mental health services for evaluation or treatment (Muriel et al., 2009). Approximately half (47%) of the oncologists indicated that they initiated a mental health referral, and a similar percentage (48%) indicated that they initiated a referral and medications, primarily antidepressants.

## Discussion

In 2005, the Canadian Strategy for Cancer Control designated emotional distress as the "sixth vital sign" and recognized its importance as an indicator of health and well-being (Rebalance Focus Action Group, 2005). As previously discussed, the NCCN and the IOM have likewise published recommendations to screen for psychological distress in cancer survivors. These national groups recognize the significance of a cancer diagnosis and its ongoing psychological impact on survivors, loved ones, and caregivers (IOM, 2007; NCCN, 2013).

As discussed previously, there are numerous studies that demonstrate the prevalence of psychological distress in patients with chronic diseases, including cancer. Psychological distress has demonstrated a role in patient outcomes, both medical and psychological, and can affect long-term cancer survivorship. There are reliable and valid instruments available to screen distress in patients, such as the NCCN’s Distress Thermometer. Despite the availability of brief, easy-to-use instruments, health-care providers do not consistently screen for distress in patients with chronic diseases, nor do they provide referrals to the transdisciplinary team.

Advanced practitioners (APs) are trained to screen for, evaluate, and intervene in physical and psychosocial issues in patients with chronic disease. In today’s health-care climate, APs are often relegated to conform to the medical model, especially in the inpatient setting with little time to screen or appropriately assess distress. In the ambulatory setting, more opportunities may exist to assess psychosocial needs, although the AP may spend much time in treatment-related visits with significant symptom management secondary to chemotherapy and metastatic disease. Therefore, APs must allocate time to screen, assess, and treat psychosocial issues. The routine addition of a screening instrument such as the NCCN’s DT is of help to identify and prioritize sources of distress.

Distress screens cost as little as the paper they are printed on, but the time involved in further assessment and discussion for cancer survivors has yet to be quantified. Electronic methods of information acquisition may be a bit more costly at first, but provide a "green" approach and are vital to interface with electronic medical records. In the busy clinical setting, extensive discussions may be difficult to accomplish with regard to staff, space, and time. It may be helpful to have the patient prioritize their most urgent psychosocial needs. Ongoing assessment, discussion, intervention, and evaluation by the multidisciplinary nursing team may ultimately be more cost-effective, given a proactive rather than reactive approach. Unresolved issues can be fully discussed at the end of active treatment in focused cancer survivorship visits.

The completion of an individual and personalized survivorship care plan provides an opportunity for the AP to focus on each patient as they complete active treatment (Lester, 2011). The survivorship care plan is a comprehensive review of the survivor’s cancer diagnosis, treatment, and medications. Survivors should, in part, be provided psychosocial information about alterations in sexual function, family and caregiver functioning, self-image issues, potential long-term side effects of treatment, existing and potential psychosocial needs, and healthy lifestyle behaviors. The survivorship care planning visit is typically at least 45 to 60 minutes in length; thus, the allotted additional time can focus on possible interventions, referrals, and a plan for follow-up care.

## Summary

It is most certain that the oncology health-care team will provide holistic and comprehensive care when the NCCN and IOM recommendations are incorporated into daily practice. This includes patient-reported distress screens at regular intervals (e.g., baseline, during treatment, and at 3-month intervals after the completion of active treatment), assessment of the level of distress and indicated sources, interventions to ameliorate the distress, and evaluation of intended interventions. It also includes survivorship care planning with a designated time to develop and discuss a personalized survivorship care plan. As these items are incorporated into daily practice, likewise the assessment of psychosocial issues will become routine with a pattern of referrals in the clinical and community settings. As the number of cancer survivors continues to increase exponentially, with extended periods disease-free or with controlled metastatic disease, clinicians must be proactive. They must confront the demons of distress with the same vigor that is targeted at the disease itself, at all stages of the cancer trajectory. 

## Acknowledgments

This work was funded by the NCCN Foundation. Joanne Lester was a recipient of the Young Investigator’s Award. Any opinions, findings, and conclusions expressed in this material are those of the author(s) and do not necessarily reflect those of the National Comprehensive Cancer Network or the NCCN Foundation.
